# Genome-Wide Identification of the GDSL Gene Family and Functional Validation of DfDACX1 in Secondary Wall Synthesis in *Dendrocalamus farinosus*

**DOI:** 10.3390/cells15151427

**Published:** 2026-08-06

**Authors:** Xin Zhao, Yanwen Zhao, Mengqiu Chen, Man Tang, Zhijian Long, Gang Xu, Ying Cao, Shanglian Hu

**Affiliations:** 1 College of Life Science and Agri-Forestry, Southwest University of Science and Technology, Mianyang 621000, China; 2 Sichuan Provincial Forestry and Grass Land Key Laboratory for Conservation and Sustainable Utilization of Bamboo Genetic Resources in Southwest of China, Mianyang 621010, China; 3 Key Laboratory of Bamboo Cultivation and Management, National Forestry and Grassland Administration, Mianyang 621000, China

**Keywords:** *Dendrocalamus farinosus*, GDSL esterase/lipase, genome-wide identification, *DfDACX1*, secondary wall synthesis

## Abstract

GDSL esterases/lipases constitute a large and functionally versatile gene family in plants, yet their systematic characterization in bamboo—perennial woody grasses with exceptionally rapid shoot elongation—remains scarce. Here, we performed a genome-wide identification of the GDSL family in allohexaploid *Dendrocalamus farinosus* and characterized the function of a candidate gene in secondary wall synthesis. A total of 265 *DfGDSL genes* were identified and classified into nine clades; rice xylan deacetylases BS1 and DARX1 fell within Clade VIII, which harbors 75 members. Chromosomal distribution, exon–intron organization, conserved motifs, and collinearity analyses indicated that whole-genome and tandem duplications drove family expansion, with purifying selection as the predominant evolutionary force. Expression profiling across tissues and shoot developmental stages pinpointed *DfGDSL58* as the sole highly expressed *OsDARX1* homolog in *D. farinosus*, showing specific upregulation during rapid elongation (50–400 cm). Its promoter contains auxin-responsive elements, and exogenous NAA treatment significantly induced its expression, with a peak at 6 h. Heterologous overexpression of *DfGDSL58* (designated *DfDACX1*) in tobacco increased plant height and basal diameter while reducing lignin and hemicellulose deposition, xylem width, and transcript levels of xylan synthase (*NtIRX9*) and lignin biosynthetic genes (*NtC4H*, *NtCOMT*, *NtCAD*), whereas cellulose content and cellulose synthase genes (*NtCESA4*, *NtCESA7*) remained largely unchanged. Collectively, these findings demonstrate that *DfDACX1* negatively regulates secondary wall thickening and promotes longitudinal growth, likely via modulating xylan deacetylation and downstream lignin biosynthesis. This study presents the first systematic characterization of the GDSL family in *D. farinosus* and identifies *DfDACX1* as a key modulator of the trade-off between cell wall deposition and rapid shoot elongation, offering mechanistic insights into bamboo’s extraordinary growth and a potential target for engineering plant architecture.

## 1. Introduction

GDSL esterases/lipases (GELPs) represent a distinct subclass of lipolytic enzymes characterized by a conserved GDSL motif at the N-terminus, which differs from the classical GXSXG motif found in most serine hydrolases [1,2]. Members of this family possess four invariant catalytic residues—Ser, Gly, Asn, and His—distributed across four conserved blocks (I–IV), and exhibit remarkable functional versatility due to their flexible active site architecture, enabling broad substrate specificity, regioselectivity, and stereoselectivity [3]. Genome-wide analyses have identified extensive GDSL gene families across the plant kingdom, including 105 members in *Arabidopsis thaliana* [3], 114 in rice (*Oryza sativa*) [4], 121 in *Brassica rapa* [5], 194 in soybean (*Glycine max*) [6], 198 in cotton (*Gossypium hirsutum*) [2], and 58 in the medicinal orchid *Dendrobium catenatum* [7]. These enzymes participate in diverse biological processes, including plant growth and development, secondary metabolite synthesis, and responses to abiotic stresses [8].

The GDSL lipase family has been increasingly implicated in cell wall remodeling and polysaccharide metabolism, particularly through xylan deacetylation. In rice, the GDSL lipases DARX1 and BS1 function as xylan deacetylases that modulate cell wall polysaccharide composition, thereby influencing plant growth and mechanical properties [9,10]. Xylan, the predominant hemicellulosic component in secondary cell walls of grasses, is heavily acetylated, and its deacetylation status directly impacts cell wall integrity, lignocellulose recalcitrance, and biomass utilization efficiency [10,11,12,13]. Beyond cell wall modification, GDSL lipases also regulate cuticle biosynthesis and stomatal development. For instance, Arabidopsis OSP1 catalyzes wax biosynthesis and stomatal outer cuticular ledge (OCL) formation, with osp1 mutants exhibiting fused stomatal OCL coverage and enhanced drought tolerance [14]. Similarly, rice WDL1, an endoplasmic reticulum-localized GDSL lipase, regulates wax biosynthesis and epidermal cell differentiation, affecting water loss rates [15]. Both OSP1 and WDL1 are representative members of plant GDSL lipases that have undergone functional specialization. Phylogenetic analyses indicate that these enzymes are not isolated outliers but belong to conserved subclades within the GDSL family, with clear orthologs identified in diverse plant species, suggesting that their functions in cuticle and epidermal development are evolutionarily conserved. Notably, these specialized functions likely arose through gene duplication and subsequent neofunctionalization, a common mechanism driving functional diversification within the GDSL family. Despite these advances, many GDSL members remain functionally uncharacterized, particularly in non-model species, highlighting the need for systematic functional studies to fully understand the biological roles of this large and versatile gene family. These findings collectively underscore the pivotal roles of GDSL lipases in coordinating cell wall architecture, cuticle properties, and plant adaptation to environmental challenges [8].

Bamboo species, as perennial woody grasses belonging to the Poaceae family, exhibit exceptionally rapid growth rates, with some species capable of growing over one meter per day during the shooting stage [16,17]. This extraordinary growth is supported by extensive cell wall biosynthesis, rapid lignification, and massive accumulation of structural carbohydrates [18,19]. The GDSL gene family has undergone significant expansion in bamboo genomes, likely driven by whole-genome duplication and tandem duplication events [20,21], which may be functionally linked to their unique biological processes such as rapid cell wall elongation, lignification, and stress adaptation [22]. However, despite the identification of GDSL genes in several model and crop species, systematic characterization of this gene family in bamboo remains limited, and the specific functions of bamboo GDSL lipases in cell wall remodeling and rapid shoot growth have not been elucidated.

In this study, we conducted a comprehensive genome-wide identification and characterization of the GDSL gene family in *Dendrocalamus farinosus*, an allohexaploid bamboo species native to China that serves as a major pulpwood and shoot-producing bamboo in Southwest China, with over 730,000 mu (approximately 48,700 hectares) of improved varieties planted and generating more than 856 million RMB (approximately 118 million USD) in additional income for farmers and enterprises [23,24]. Through phylogenetic, structural, and expression analyses, we identified *DfGDSL58* as a homolog of rice *OsDARX1* [9,25] with specific and high expression during rapid shoot elongation. Promoter analysis revealed auxin-responsive elements in *DfGDSL58* [26], and its expression was significantly induced by NAA treatment. Heterologous overexpression of *DfGDSL58* (designated *DfDACX1*) in tobacco promoted longitudinal growth while reducing lignin and hemicellulose deposition, accompanied by downregulation of secondary wall biosynthetic genes. These findings reveal that *DfDACX1* negatively regulates secondary wall thickening and promotes longitudinal growth, highlighting a novel role for a GDSL lipase in coordinating cell wall remodeling with the extraordinary shoot elongation rates characteristic of bamboo.

## 2. Materials and Methods

### 2.1. Identification of the GDSL Gene Family in D. farinosus

To comprehensively identify members of the GDSL gene family in *D. farinosus*, the known GDSL protein sequences from *Arabidopsis thaliana* (105 sequences) and rice (*Oryza sativa*, 114 sequences) were used as query probes. These sequences were retrieved from The Arabidopsis Information Resource (TAIR, https://www.arabidopsis.org/) and the rice genome database (Phytozome13.0, https://phytozome-next.jgi.doe.gov/pz/portal.html), respectively, and were employed to search against the *D. farinosus* genome using BLASTP (https://blast.ncbi.nlm.nih.gov/Blast.cgi?PAGE=Proteins) with an E-value threshold of 1 × 10^−10^.

To ensure the accuracy of the identified candidates, a complementary hidden Markov model (HMM) search was performed. The GDSL lipase conserved domain profile (PF00657) was obtained from the Pfam database (http://pfam.xfam.org/), and HMMER scanning was conducted with a cutoff value of 0.01. Candidate genes containing the complete PF00657 domain were further verified using the Conserved Domain Database (CDD) at NCBI. Subsequently, redundant and incomplete sequences were removed, resulting in the final identification of 265 GDSL genes in *D. farinosus*.

For each identified GDSL gene, the corresponding mRNA, protein, and genomic sequences, along with annotation data, were extracted from the *D. farinosus* genome database using TBtools software (v2.340, developed by Chen et al., 2020, available at https://github.com/CJ-Chen/TBtools) [25]. Basic physicochemical properties of the deduced GDSL proteins, including amino acid length, theoretical isoelectric points (pI), and molecular weights (MW), were calculated using the ExPASy online tool (https://www.expasy.org). To investigate the evolutionary relationships among GDSL lipases from *D. farinosus*, rice, and *A. thaliana*, multiple sequence alignments of the full-length protein sequences were performed using ClustalW (https://www.genome.jp/tools-bin/clustalw). A maximum likelihood (ML) phylogenetic tree was then constructed using MEGA11 (Molecular Evolutionary Genetics Analysis) software, with the Jones–Taylor–Thornton (JTT) model of amino acid substitution. Branch support was assessed using bootstrap analysis with 1000 replicates. The resulting phylogenetic tree was visualized and annotated using the iTOL online tool (https://itol.embl.de).

### 2.2. Chromosomal Localization, Gene Structure, and Conserved Motif Analysis

To determine the chromosomal distribution of *DfGDSL* genes, the gene position information was extracted from the *D. farinosus* genome annotation file, and chromosomal localization was visualized using TBtools software (v2.340). The exon–intron structures of *DfGDSL* genes were analyzed using the Gene Structure Display Server (GSDS 2.0, http://gsds.gao-lab.org/) based on the coding sequences and corresponding genomic sequences. Conserved motifs within the DfGDSL proteins were identified using the MEME online tool (https://meme-suite.org/meme/index.html) with the following parameters: maximum number of motifs set to 10 and motif width ranging from 6 to 50 amino acids. The conserved domains were further confirmed through searches against the NCBI Conserved Domain Database (CDD, https://www.ncbi.nlm.nih.gov/cdd/). The resulting phylogenetic trees, gene structures, and motif distributions were integrated and visualized using TBtools.

### 2.3. Promoter Cis-Acting Element Analysis

To investigate the potential transcriptional regulatory mechanisms of *DfGDSL* genes, the 2.0 kb genomic sequences immediately upstream of the start codon (ATG) of each *DfGDSL* gene were extracted from the *D. farinosus* genome using TBtools (v2.340). These putative promoter sequences were then submitted to the PlantCARE online database (http://bioinformatics.psb.ugent.be/webtools/plantcare/html/) for cis-acting regulatory element prediction. The identified cis-elements were categorized based on their reported functions, and their distribution patterns were visualized using TBtools (v2.340).

### 2.4. Plant Materials and Stress Treatments

*D. farinosus* plants were cultivated under natural conditions at the bamboo germplasm resource garden of Southwest University of Science and Technology (Mianyang, Sichuan, China). For tissue-specific expression analysis, samples of roots, stems, and leaves were collected from mature plants. For bamboo shoot developmental stage analysis, bamboo shoots at different heights (10 cm, 50 cm, 100 cm, 200 cm, 300 cm, and 400 cm) were collected. Additionally, to examine the response of candidate genes to auxin treatment, leaves of *D. farinosus* were treated with 10 µM NAA (1-naphthaleneacetic acid) solution, and samples were collected at 0, 1, 6, and 12 h after treatment. All samples were immediately frozen in liquid nitrogen and stored at −80 °C until further use. Three biological replicates were performed for each treatment.

### 2.5. RNA Extraction and Quantitative Real-Time PCR (qRT-PCR) Analysis

Total RNA was extracted from the collected *D. farinosus* tissues using an RNAprep Pure Plant Plus Kit (Tiangen, Beijing, China) following the manufacturer’s instructions. RNA quality and concentration were assessed using agarose gel electrophoresis and a NanoDrop 2000 spectrophotometer (Thermo Scientific, Waltham, MA, USA). First-strand cDNA was synthesized from 1 µg of total RNA using a PrimeScript™ RT Reagent Kit with gDNA Eraser (Takara, Dalian, China). Gene-specific primers for selected *DfGDSL* genes were designed using Primer Premier 5.0 software, and their specificity was validated through melting curve analysis. qRT-PCR was performed on a CFX96 Real-Time PCR Detection System (Bio-Rad, Hercules, CA, USA) using TB Green Premix Ex Taq™ II (Takara, Dalian, China) with the following thermal cycling conditions: 95 °C for 30 s, followed by 40 cycles of 95 °C for 5 s and 60 °C for 30 s. The *DfActin* gene was used as the internal reference gene. Relative expression levels were calculated using the 2^−ΔΔCT^ method. Three technical replicates were conducted for each biological replicate.

### 2.6. Vector Construction and Heterologous Transformation of Tobacco

To investigate the biological function of *DfGDSL58*, the full-length coding sequence (CDS) of *DfGDSL58* was amplified from *D. farinosus* cDNA using gene-specific primers containing appropriate restriction enzyme sites. The amplified fragment was cloned into the plant binary overexpression vector pCAMBIA1300, driven by the CaMV 35S promoter, to generate the recombinant construct 35S::*DfGDSL58*. The construct was verified by Sanger sequencing and then introduced into *Agrobacterium tumefaciens* strain GV3101 competent cells using the freeze–thaw method. *Nicotiana tabacum* (tobacco) transformation was performed via the leaf disc method. Transgenic plants were selected on Murashige and Skoog (MS) medium containing 50 mg/L kanamycin. The integration and expression of *DfGDSL58* in transgenic lines were confirmed by PCR and qRT-PCR, respectively. Three independent transgenic lines (OE1–OE3) with elevated expression levels were selected for phenotypic analysis.

### 2.7. Phenotypic Analysis and Cell Wall Component Measurement

The height and basal diameter of wild-type (WT) and transgenic tobacco plants were measured at 15 and 35 days after transplantation. For anatomical observation, the 6th internode of tobacco stems was collected and fixed in FAA solution (formaldehyde:acetic acid:70% ethanol = 1:1:18). Transverse sections (10 µm thickness) were prepared using a rotary microtome and stained with phloroglucinol–HCl solution (1% phloroglucinol in 6 M HCl) for 5 min to visualize lignin deposition. Stained sections were observed and photographed under a light microscope, and xylem width was measured using ImageJ (https://imagej.net/ij/) software. For cell wall component analysis, stems of 35-day-old tobacco plants were dried at 65 °C to constant weight and ground to pass through a 40-mesh sieve. The ground samples were extracted with a benzene/ethanol mixture (2:1, *v*/*v*) for 8 h using a Soxhlet apparatus to remove extractives, followed by air-drying.

Lignin content was determined by the Klason method according to standard procedures [27]. Approximately 0.2 g of extractive-free sample was hydrolyzed with 3 mL of 72% (*w*/*w*) H_2_SO_4_ at 20 °C for 2 h with occasional stirring. The mixture was then diluted with 100 mL of distilled water and autoclaved at 121 °C for 1 h. The resulting acid-insoluble residue was collected by filtration through a pre-weighed fine-porosity sintered glass crucible, washed thoroughly with hot water until neutral, and dried at 105 °C to constant weight. Acid-insoluble lignin (Klason lignin) was determined gravimetrically by weighing the dried residue. Acid-soluble lignin was determined spectrophotometrically using the acetyl bromide method as previously described, with absorbance measured at 280 nm. Total lignin content was calculated as the sum of acid-insoluble and acid-soluble lignin fractions [28,29]. Cellulose content was determined gravimetrically following the Van Soest detergent fiber method. Briefly, the extractive-free sample was sequentially treated with neutral detergent solution, acid detergent solution, and 72% H_2_SO_4_. The final residue after acid hydrolysis was dried and weighed as cellulose content. For hemicellulose content, the difference between the acid detergent fiber (ADF) residue and the acid-insoluble lignin residue was calculated, representing the hemicellulose fraction [30]. All measurements were performed with three biological replicates, and results were expressed as percentage of the dry weight of extractive-free sample.

### 2.8. RNA Extraction and qRT-PCR Analysis of Transgenic Tobacco Lines

Total RNA was extracted from leaves and stems of WT and transgenic tobacco plants using the RNAprep Pure Plant Plus Kit (Tiangen, Beijing, China). cDNA synthesis and qRT-PCR were performed as described in Section 2.5. The expression levels of cell wall biosynthesis-related genes, including *NtIRX9* (xylan synthase), *NtCESA4* and *NtCESA7* (cellulose synthases), and lignin biosynthesis genes (*NtPAL*, *NtC4H*, *NtCOMT*, and *NtCAD*), were examined. The *NtActin* gene was used as the internal reference. Three biological replicates were performed for each line.

### 2.9. Statistical Analysis

All data are presented as means ± standard deviation (SD) from at least three biological replicates. Statistical significance was determined by Student’s *t*-test or one-way ANOVA using graphpad prism software (version 10.0). Differences were considered statistically significant at * *p* < 0.05, and highly significant at ** *p* < 0.01. Graphs were generated using GraphPad Prism (version 10.0) or TBtools (version 2.340).

## 3. Results

### 3.1. Identification and Phylogenetic Analysis of the D. farinosus GDSL Gene Family

**Figure 1:**
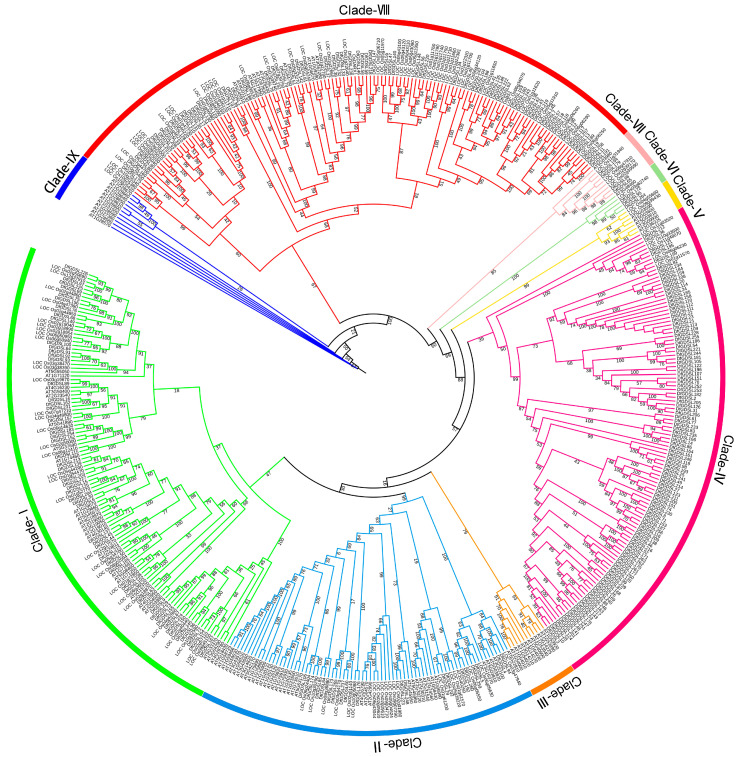
Phylogenetic analysis of the GDSL gene family in *D. farinosus*, rice, and *Arabidopsis thaliana.* A maximum likelihood (ML) phylogenetic tree was constructed based on the full-length amino acid sequences of GDSL proteins from *D. farinosus* (265 proteins), rice (114 proteins), and *A. thaliana* (105 proteins). The tree was divided into nine distinct clades (I–IX), as indicated by different background colors, based on the topological structure and bootstrap support from the ML analysis. Bootstrap values (1000 replicates) are shown at the branch nodes, with values ≥ 50% indicated; higher values represent stronger statistical support for the branching topology. The functionally characterized rice xylan deacetylases BS1 and DARX1 are highlighted in bold and located in Clade VIII. The scale bar at the bottom represents the evolutionary distance (substitutions per site).

To systematically identify GDSL family members in *D. farinosus*, we used 105 *Arabidopsis thaliana* and 114 rice GDSL protein sequences as query probes against the *D. farinosus* genome. Initial screening with the GDSL conserved domain (PF00657) yielded 309 candidate genes (Supplementary Table S1). After removing redundant and incomplete sequences through conserved domain alignment against the NCBI database, a final set of 265 high-confidence GDSL genes was retained, designated as *DfGDSL1* through *DfGDSL265*, including all three subgenomes (A, B, and C). (Supplementary Table S2). To elucidate the evolutionary relationships of DfGDSL lipases, we constructed a maximum likelihood (ML) phylogenetic tree using full-length protein sequences from *A. thaliana*, rice, and *D. farinosus* (Figure 1). The 265 DfGDSL proteins were unevenly distributed across nine distinct clades (Clades I–IX) (Figure 1). Clade IV contained the largest number of members (111), followed by Clade VIII with 75 members, whereas Clades III and IX lacked any DfGDSL representative. Notably, the functionally characterized rice xylan deacetylases BS1 and DARX1 were positioned within Clade VIII, implying that DfGDSL members within this clade may also be involved in cell wall polysaccharide deacetylation.

### 3.2. Chromosomal Allocation, Gene Structure, and Motif Identification

**Figure 2:**
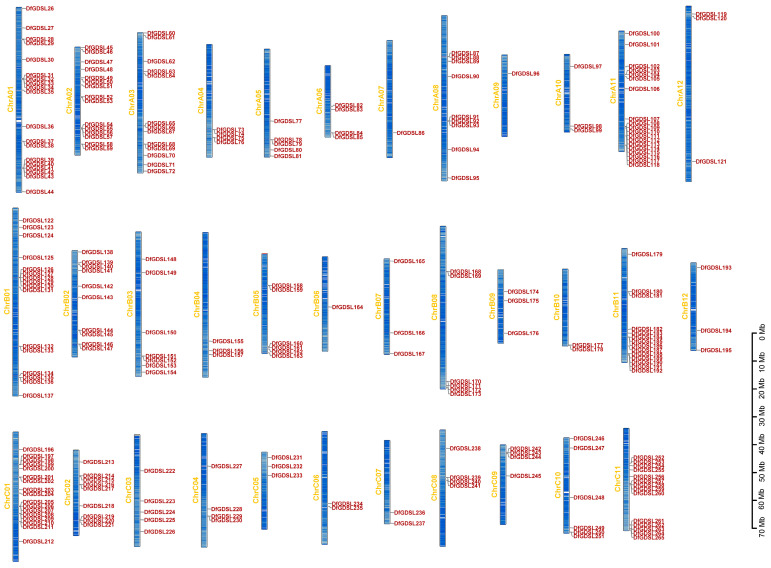
Chromosomal localization of *D. farinosus* GDSL genes. The 265 *DfGDSL genes* were mapped to the 35 chromosomes of the *D. farinosus* genome. Chromosome numbers are labeled in blue on the left side of each chromosome. The chromosome nomenclature A, B, and C denotes the three subgenomes of this allohexaploid species, with numbers indicating chromosome number within each subgenome. *DfGDSL gene* names are marked in dark red on the right side of each chromosome. The scale bars on the left indicate chromosome sizes in megabases (Mb).

The 265 *DfGDSL* genes were designated as *DfGDSL1* through *DfGDSL265* according to their chromosomal positions (Supplementary Table S2). Their predicted protein properties, including isoelectric points and molecular weights, were calculated (Supplementary Table S3), and subcellular localization analysis using WoLF PSORT (https://wolfpsort.hgc.jp) revealed a broad distribution across multiple compartments, including the nucleus, cytoplasm, chloroplast, vacuole, mitochondria, and Golgi apparatus (Supplementary Table S4). Chromosomal mapping showed that the 265 genes were widely but unevenly distributed across all 35 chromosomes (Figure 2). The chromosome nomenclature A, B, and C denotes the three subgenomes of this allohexaploid species, with numbers indicating chromosome number within each subgenome. Clustered gene loci were observed on chromosomes A01 (19 genes), B01 (16), C01 (17), A11 (19), B11 (14), and C11 (14), whereas chromosomes A07, A09, and B06 each contained only a single *DfGDSL* gene. To further investigate the nature of these clustered loci, we examined the genomic organization and gene orientation of *DfGDSL* members within the six major clustered regions (A01, B01, C01, A11, B11, and C11). Adjacent gene pairs within these clusters were predominantly separated by intergenic regions of less than 10 kb, a hallmark feature of tandem duplication events, suggesting that tandem duplications have contributed substantially to the local expansion of the *DfGDSL* family in these chromosomal regions. Analysis of gene orientation revealed diverse configurations within the clusters, including head-to-tail (convergent), tail-to-head (divergent), and head-to-head arrangements. Notably, several gene pairs in the A01 and C01 clusters were arranged in a head-to-head orientation with intergenic regions of less than 2 kb, raising the possibility that they may share bidirectional promoters for coordinated transcriptional regulation. However, systematic validation of promoter sharing and co-expression patterns would require additional experimental approaches. Collectively, these observations suggest that tandem duplication, followed by potential functional divergence, has played a significant role in shaping the genomic architecture of the *DfGDSL* family in *D. farinosus* [31]. As an allohexaploid species, *D. farinosus* harbors substantially more GDSL genes (265) than *Arabidopsis thaliana* (105) and rice (114), suggesting that whole-genome duplication and tandem duplication events have contributed significantly to the expansion of this family in bamboo, potentially in association with unique biological processes such as rapid cell wall deposition, lignification, and stress adaptation.

**Figure 3:**
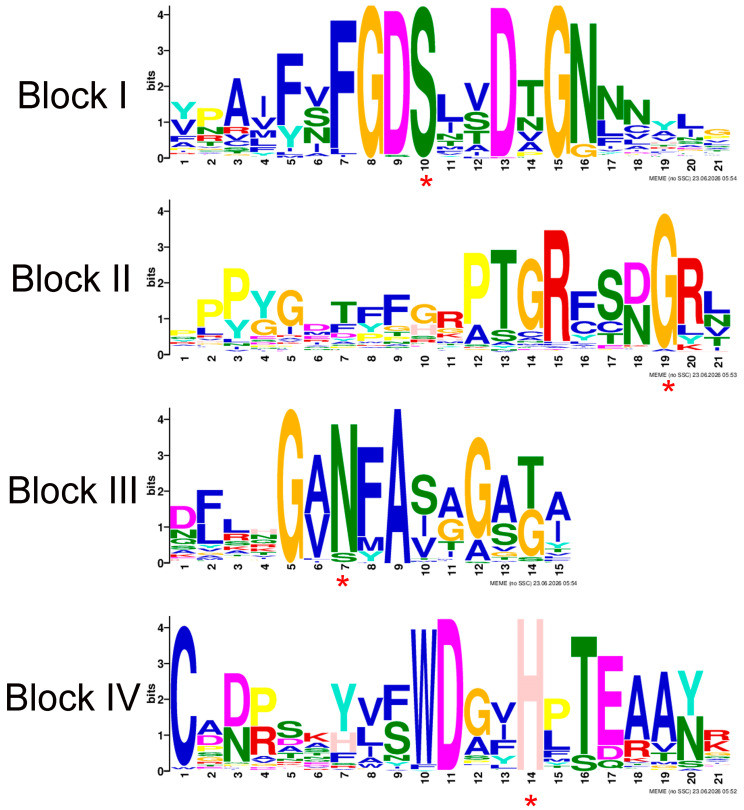
Motif logos of four conserved regions detected in the conserved domain prediction of all DfGDSL proteins in *D. farinosus*. The four conserved blocks (I, II, III, and IV) of the GDSL family were identified using MEME motif analysis (https://meme-suite.org/meme/index.html). The height of each letter represents the relative frequency of the corresponding amino acid at that position. The conserved catalytic residues—serine (S), glycine (G), asparagine (N), and histidine (H)—are marked with red asterisks. These four residues are invariant across the GDSL family and are essential for lipase/esterase activity.

Analysis of exon–intron structures revealed that the intron number among *DfGDSL* genes ranged from 1 to 9 (Supplementary Table S5). *DfGDSL65* lacked introns, whereas *DfGDSL74* contained the highest number (9). The most frequent configuration was four introns, found in 124 members (46.8% of the total), followed by three introns (48 members, 18.1%), five introns (31, 11.7%), one intron (29, 10.9%), and two introns (23, 8.7%). Members with six, seven, or eight introns were relatively rare, and the nine-intron configuration was unique to *DfGDSL74*. Furthermore, ten conserved motifs were identified in the DfGDSL protein family (Supplementary Figure S1). Among these, Motifs 2, 3, 6, and 8 were present in nearly all members (Figure 3), corresponding to the conserved Blocks I, II, III, and IV of the GDSL family, respectively, implying their essential roles in maintaining protein structure and function.

**Figure 4:**
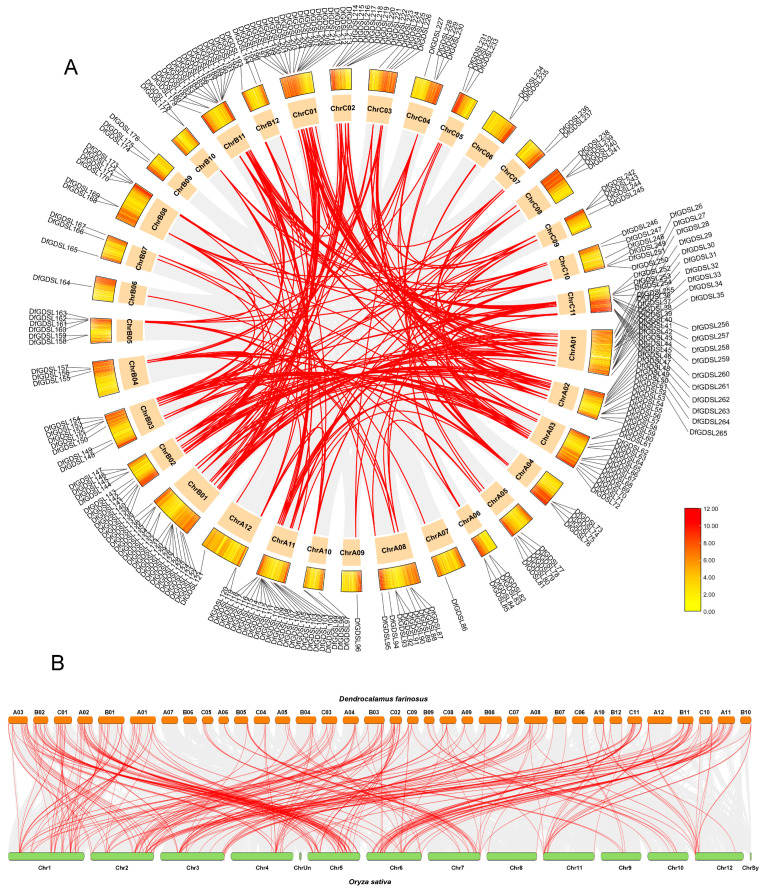
Intraspecific (**A**) and interspecific collinearity analysis with rice (**B**) of DfGDSLs in *D. farinosus.* (**A**) Intraspecific collinearity analysis showing 228 collinear gene pairs within the *D. farinosus* genome. Red lines connect homologous gene pairs between different chromosomes. (**B**) Interspecific collinearity analysis between *D. farinosus* and rice (*Oryza sativa*), revealing 277 collinear gene pairs. Red lines represent syntenic relationships between the two species. Chromosome designations: A, B, C denote the three subgenomes of *D. farinosus*; Os denotes rice chromosomes.

Intraspecific collinearity analysis identified 228 collinear gene pairs within the *D. farinosus* genome (Figure 4A, Supplementary Table S6). The Ka/Ks ratios for all these pairs were below 1, indicating that the *DfGDSL* family has predominantly undergone purifying selection during evolution, with functional constraints maintaining their conserved roles. Interspecific collinearity analysis, using rice as a reference genome, revealed 277 collinear gene pairs between 114 rice GDSL genes and 265 *D. farinosus* GDSL genes (Figure 4B). This number substantially exceeds that of intraspecific collinear pairs, suggesting that extensive gene expansion occurred following species divergence, which may represent an evolutionary strategy enabling bamboo to adapt to specific ecological niches or acquire diverse biological functions.

### 3.3. Tissue-Specific Expression and Developmental Dynamics of DfGDSL Genes

To gain initial insights into the biological functions of the *DfGDSL* gene family, we generated expression heatmaps based on transcriptome data from roots, stems, leaves, and bamboo shoots at various developmental stages (Supplementary Figure S2 and Supplementary Table S7). Tissue-specific expression profiling revealed distinct preferences among family members: *DfGDSL62*, *DfGDSL222*, and *DfGDSL262* were preferentially expressed in roots; *DfGDSL262*, *DfGDSL240*, *DfGDSL222*, *DfGDSL169*, *DfGDSL58*, and *DfGDSL56* showed high transcript abundance in stems; and *DfGDSL257* and *DfGDSL242* were predominantly expressed in leaves, suggesting that different *DfGDSL* members may contribute to organ-specific developmental regulation. During the rapid elongation phase of bamboo shoots (from 50 cm to 400 cm in height), several genes, including *DfGDSL58*, *DfGDSL56*, *DfGDSL120*, *DfGDSL169*, *DfGDSL262*, and *DfGDSL239*, were significantly upregulated, implicating them in internode elongation and the rapid growth process.

**Figure 5:**
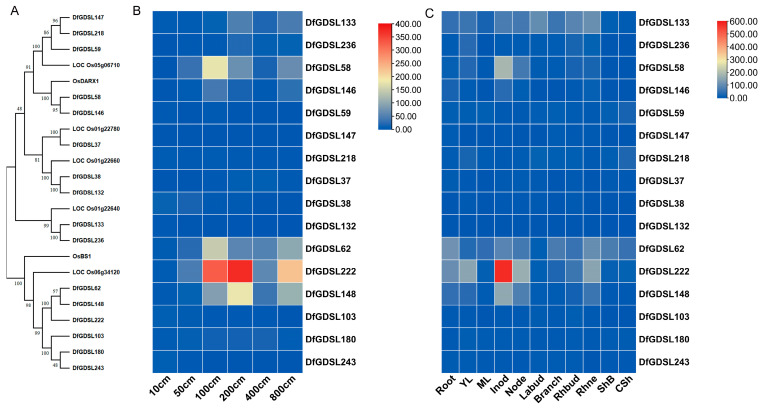
Phylogenetic tree and expression profile analysis of OsDARX1/OsBS1 homologous genes in *D. farinosus* DfGDSLs. (**A**) Phylogenetic clade of *D. farinosus* DfGDSLs with rice BS1 and DARX1. The clade was extracted from the full ML tree shown in Figure 1. *DfGDSL* members within this clade are shown in different colors, with the rice *BS1* and *DARX1* highlighted in red. (**B**) Expression levels (FPKM values) of *D. farinosus GDSL homologous genes* of *OsBS1* and *OsDARX1* in bamboo shoots at different growth stages (10 cm, 50 cm, 100 cm, 200 cm, 300 cm, and 400 cm in height). FPKM (Fragments Per Kilobase of transcript per Million mapped reads) values were derived from RNA-seq data. (**C**) Expression levels (FPKM values) of the same genes in different tissues, including root, young leaf (YL). mature leaf (ML), branch, culm sheath (CSh), internode (Inod), lateral bud (Labud), node, Rhizome bud (Rhbud), rhizome neck (Rhne), bamboo shoot sheath (ShB). Data are presented as mean FPKM values from three biological replicates. See Supplementary Table S7 for detailed data.

Given that rice *DARX1* and *BS1* encode xylan deacetylases involved in cell wall remodeling and growth regulation, we focused on the phylogenetic clade containing their *D. farinosus* homologs and examined their expression profiles in detail (Figure 5A). The *OsBS1* homologs (*DfGDSL222*, *DfGDSL62*, and *DfGDSL148*) exhibited high and relatively coordinated expression in both stems and rapidly elongating shoots, suggesting possible functional redundancy. In contrast, the *OsDARX1* homolog *DfGDSL58* displayed markedly elevated expression in bamboo shoots, significantly exceeding its levels in other tissues (Figure 5B,C), strongly indicating a specific association with shoot development and a potential role as a key regulator of rapid elongation.

**Figure 6:**
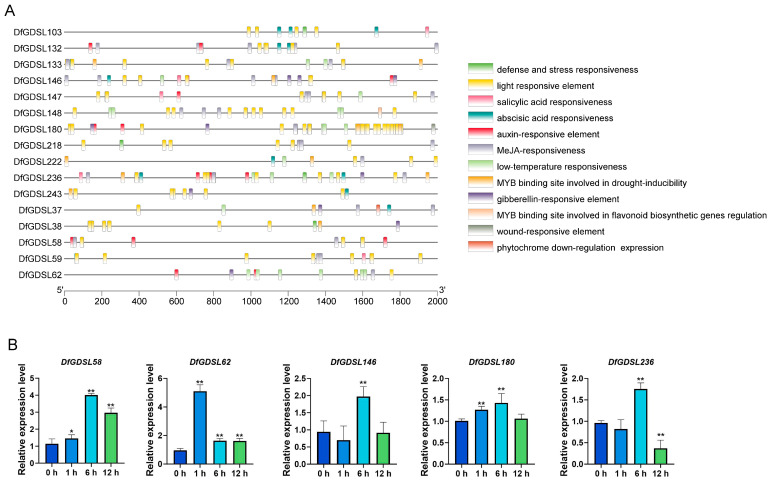
Prediction of promoter cis-acting elements and qRT-PCR validation of *OsDARX1/OsBS1* homologous genes in *D. farinosus DfGDSLs*. (**A**) Prediction of cis-acting elements in the promoters of 16 *D. farinosus DfGDSL homologous genes* of *OsDARX1/OsBS1*. The 2.0 kb upstream promoter regions were analyzed using PlantCARE. Different colored boxes represent different types of cis-acting regulatory elements: plant hormone-responsive (ABRE, CGTCA-motif, TGACG-motif, TGA-box, TGA-element, AuxRR-core, SARE, TCA-element, GARE-motif, P-box, TATC-box), light-responsive, and stress-inducible elements (drought-inducible MYB-binding sites, low-temperature responsive elements). The number of each element type is shown in the color legend. (**B**) qRT-PCR validation of 5 genes containing auxin-responsive elements (*DfGDSL58*, *DfGDSL62*, *DfGDSL146*, *DfGDSL180*, and *DfGDSL236*). *D. farinosus* leaves were treated with 100 µM NAA, and samples were collected at 0 h, 1 h, 6 h, and 12 h post-treatment. Relative expression levels were calculated using the 2^−ΔΔCT^ method with *DfTublin* as the internal reference gene. Values are means ± SD from three biological replicates. Asterisks indicate significant differences compared to 0 h (* *p* < 0.05, ** *p* < 0.01; one-way ANOVA with Tukey’s HSD test).

To explore the transcriptional regulation of the 16 *OsDARX1/OsBS1* homologous *DfGDSL* genes (Figure 6A), we analyzed their 2 kb upstream promoter regions using PlantCARE. The promoters were enriched with diverse cis-acting elements, including phytohormone-responsive motifs (ABRE, CGTCA-motif/TGACG-motif, TGA-box/TGA-element/AuxRR-core, SARE/TCA-element, and GARE-motif/P-box/TATC-box), light-responsive elements, and stress-inducible elements (drought-inducible MYB-binding sites and low-temperature-responsive elements). Light-responsive elements were the most abundant and present in nearly all promoters, implying widespread involvement of these genes in light signaling networks. Notably, six promoters—including that of *DfGDSL58*—harbored at least two auxin-responsive elements, suggesting potential regulation by auxin signaling during bamboo shoot growth. Additionally, promoters of *DfGDSL103*, *DfGDSL132*, *DfGDSL148*, and *DfGDSL62* contained multiple stress-responsive elements, hinting at their possible roles in stress adaptation.

Based on the promoter analysis, we selected five auxin-responsive element-containing genes (*DfGDSL58*, *DfGDSL62*, *DfGDSL146*, *DfGDSL180*, and *DfGDSL236*) and examined their expression dynamics in *D. farinosus* leaves following exogenous NAA treatment using qRT-PCR (Figure 6B). Among them, *DfGDSL58* showed the most significant and temporally regular induction, peaking at 6 h post-treatment. Given its specific and high expression in bamboo shoots, its continuous upregulation during rapid shoot elongation (50–400 cm), and its status as the sole highly expressed *OsDARX1* homolog in *D. farinosus*, we designated *DfGDSL58* as *DfDACX1* (*D. farinosus* Deacetylase on Xylan 1) and selected it for subsequent functional characterization to investigate its role in rapid shoot growth and cell wall modification.

Although *DfGDSL58, DfGDSL62, DfGDSL146, DfGDSL180*, and *DfGDSL236* all contain auxin-responsive elements in their promoters, their expression kinetics following NAA treatment exhibited notable differences in both induction amplitude and peak timing. This variation may be attributed to several non-exclusive factors: differences in the number, exact position, or spacing of auxin-responsive elements within each promoter; the presence of additional cis-regulatory motifs that modulate or fine-tune auxin responsiveness; or inherent differences in transcript stability and turnover rates among these genes. Collectively, these observations suggest that while auxin signaling broadly activates multiple *DfGDSL* members, the precise regulatory output is refined by promoter architecture and gene-specific features.

### 3.4. Heterologous Overexpression of DfGDSL58 in Tobacco Promotes Plant Growth Rate and Reduces Cell Wall Thickening

**Figure 7:**
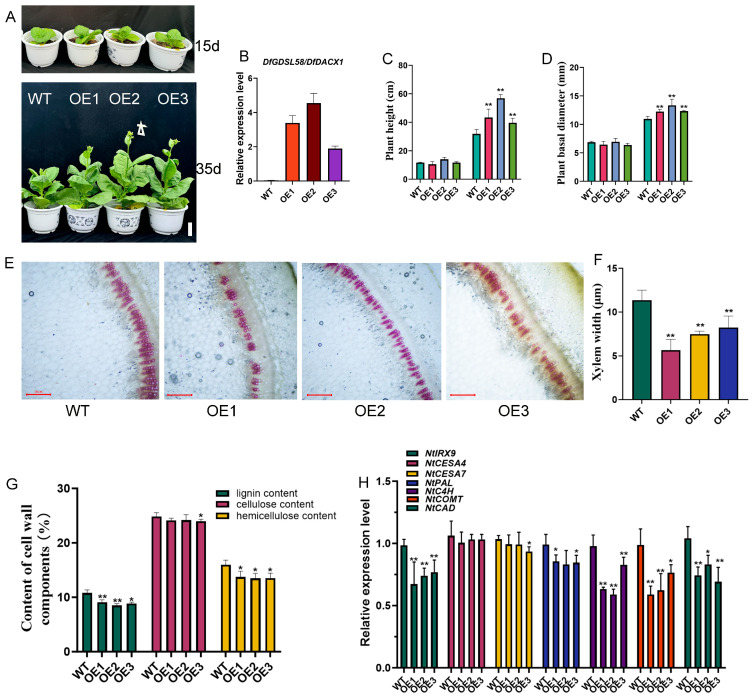
Phenotypic analysis of *DfGDSL58*-overexpressing tobacco plants. (**A**) Overall phenotype of wild-type (WT) and *DfGDSL58*-overexpressing tobacco lines (OE1–OE3) at 35 days after transplantation. (**B**) Relative expression levels of *DfGDSL58* in WT and OE lines, determined by qRT-PCR with *NtActin* as the internal reference. (**C**) Plant height measurements of WT and OE lines at 15 and 35 days after transplantation. (**D**) Basal diameter measurements at 15 and 35 days after transplantation. (**E**) Phloroglucinol–HCl staining of transverse sections of the 6th internode; lignin deposition appears as red coloration. Scale bar = 20 µm. (**F**) Xylem width measurements from stained sections. (**G**) Cell wall component contents (cellulose, lignin, and hemicellulose) in stems of WT and OE lines, expressed as percentage of dry weight. (**H**) Relative expression levels of cell wall biosynthesis-related genes (*NtIRX9*, *NtCESA4*, *NtCESA7*, *NtPAL*, *NtC4H*, *NtCOMT*, and *NtCAD*) in WT and OE lines, determined by qRT-PCR with *NtActin* as the internal reference. All values are means ± SD from three biological replicates (*n* = 3 per line). Statistical significance was determined by one-way ANOVA with Tukey’s HSD test (* *p* < 0.05, ** *p* < 0.01).

To functionally characterize *DfGDSL58* (designated *DfDACX1*), we generated stable transgenic tobacco lines through *Agrobacterium*-mediated transformation using a 35S-driven overexpression construct (Figure 7A,B). Compared with wild-type (WT) plants, three independent overexpression lines (OE1–OE3) exhibited significantly increased plant height and basal diameter (Figure 7C,D), indicating that *DfDACX1* overexpression enhances overall vegetative growth. To determine whether the increase in stem thickness was accompanied by alterations in vascular development, we performed paraffin sectioning and phloroglucinol staining on the 6th internode of transgenic plants. Overexpression lines displayed markedly weaker lignin staining signals and significantly reduced xylem width relative to WT (Figure 7E,F), suggesting that *DfDACX1* negatively regulates lignin deposition and xylem development. Quantitative analysis of cell wall components further confirmed that lignin and hemicellulose contents were significantly decreased in stems of overexpression lines, whereas cellulose content exhibited only a marginal, non-significant reduction (Figure 7G).

Consistent with these biochemical changes, qRT-PCR analysis revealed that the expression of the xylan synthase gene *NtIRX9* and lignin biosynthesis genes *NtC4H*, *NtCOMT*, and *NtCAD* were significantly downregulated in overexpression lines, while the cellulose synthase genes *NtCESA4* and *NtCESA7* showed no substantial expression changes (Figure 7H). This transcriptional profile closely paralleled the observed changes in cell wall composition. Collectively, these results suggest that *DfDACX1* overexpression promotes longitudinal growth by suppressing lignin and hemicellulose biosynthesis, thereby reducing secondary wall accumulation while potentially reallocating carbon and energy resources toward vertical elongation. The moderate reduction in lignin content, without a corresponding decrease in cellulose, implies that the stem mechanical integrity may be maintained, although direct mechanical testing would be required for definitive confirmation, implying that *DfDACX1* may serve as a modulator of the balance between cell wall thickening and vegetative growth.

## 4. Discussion

In this study, we performed a comprehensive genome-wide identification of the GDSL gene family in *D. farinosus*, an allohexaploid bamboo species with significant economic and ecological importance. A total of 265 *DfGDSL genes* were identified, substantially exceeding the numbers reported in *Arabidopsis thaliana* (105) [3], rice (114) [4], and even cotton (198) [2] (Figure 1 and Supplementary Figure S1). These 265 genes represent the sum of all gene copies present across the three subgenomes (A, B, and C) of the allohexaploid genome. Collinearity analysis using MCScanX revealed extensive collinear gene pairs among the A, B, and C subgenomes (e.g., A01–B01–C01, A11–B11–C11), indicating that most *DfGDSL genes* are conserved across subgenomes, with copy number variations reflecting gene loss and gain events following allopolyploidization (Figure 4). This remarkable expansion is likely attributable to the allohexaploid nature of *D. farinosus* and the extensive whole-genome duplication and tandem duplication events that have occurred throughout bamboo evolution [22,32]. The uneven distribution of *DfGDSL genes* across the 35 chromosomes, with evident clustering on several chromosomes (e.g., A01, B01, C01, A11), further supports the contribution of tandem duplications to family expansion [2]. Notably, the Ka/Ks ratios of all intraspecific collinear gene pairs were less than 1, indicating that purifying selection has predominantly shaped the evolution of this family, preserving its core functions [6]. The identification of 277 interspecific collinear gene pairs between *D. farinosus* and rice far exceeded the 228 intraspecific pairs, suggesting that extensive gene expansion occurred after species divergence, which may represent an important evolutionary strategy for bamboo to adapt to its unique ecological niches and developmental programs [22,33,34]. Regarding whether the observed *GDSL* family size (265 members) is typical for *D. farinosus*, a systematic comparison across all gene families in this allohexaploid genome would be required to establish a genome-wide baseline distribution, which is beyond the scope of the current study. Nevertheless, the *GDSL* family size in *D. farinosus* (265) is comparable to that reported in other polyploid species, such as cotton (198 in allotetraploid *G. hirsutum*) [2] and soybean (194 in paleopolyploid *G. max*) [6], suggesting that the observed expansion is consistent with the general trend of GDSL family enlargement following polyploidization events. Future genome-wide bootstrapping analyses would be valuable to statistically assess whether the size of the GDSL family deviates from random expectations across the entire gene family landscape. The clustering of *DfGDSL genes* on specific chromosomal regions, particularly on chromosomes A01, B01, C01, A11, B11, and C11, is consistent with the notion that the physical proximity of functionally related genes facilitates coordinated transcriptional regulation. Such genomic organization has been shown to be evolutionarily conserved across eukaryotes, where adjacent gene pairs exhibit tighter co-regulation than unpaired genes [31]. Furthermore, the transcriptional coordination of clustered genes often depends on chromatin remodelers that modulate local chromatin accessibility [35]. The clustering of metabolically related genes is also conserved across diverse fungal lineages, suggesting functional significance beyond mere genomic proximity [36]. In the context of the *DfGDSL* family, the observed clustering may facilitate the coordinated expression of multiple *GDSL* members in response to developmental or environmental cues, potentially contributing to the dynamic regulation of cell wall remodeling during bamboo shoot elongation. However, direct evidence for such coordinated regulation would require comparative expression analysis of clustered versus non-clustered *DfGDSL genes*, as well as chromatin accessibility profiling, which remain important directions for future research.

The phylogenetic classification of the 265 *DfGDSL genes* into nine clades revealed that the functionally characterized rice xylan deacetylases BS1 and DARX1 [9,10] are located within Clade VIII. This clade contains 75 *DfGDSL* members, suggesting that bamboo may possess an expanded repertoire of genes potentially involved in cell wall polysaccharide deacetylation. Given that bamboo cell walls are characterized by high hemicellulose content and rapid lignification during shoot elongation [18,37,38], the expansion of *GDSL genes* within this clade may be functionally relevant to the dynamic cell wall remodeling required for rapid growth. Consistent with this hypothesis, our tissue-specific expression profiling revealed that several Clade VIII members, including *DfGDSL58*, *DfGDSL62*, *DfGDSL222*, and *DfGDSL148*, exhibited high expression in stems and during the rapid elongation phase of bamboo shoots (50 cm to 400 cm) (Figure 4 and Figure 5). Among these, *DfGDSL58*—the sole highly expressed homolog of rice *OsDARX1* in *D. farinosus*—displayed particularly prominent and specific expression in bamboo shoots, strongly implicating it in the regulation of rapid shoot growth.

Promoter cis-element analysis of the 16 *OsDARX1*/*OsBS1* homologous genes provided further insights into their potential regulatory mechanisms. The widespread presence of light-responsive elements and phytohormone-related cis-elements, including ABA, MeJA, auxin, SA, and GA response elements, suggests that these genes are subject to complex transcriptional regulation integrating multiple developmental and environmental signals [26] (Figure 6). Notably, six genes, including *DfGDSL58*, contained at least two auxin-responsive elements in their promoters, and exogenous NAA treatment significantly induced *DfGDSL58* expression, with peak induction at 6 h. This auxin responsiveness is particularly intriguing given the established role of auxin in promoting bamboo internode elongation [16,39,40]. The combination of auxin inducibility, shoot-specific high expression, and phylogenetic relatedness to *OsDARX1* led us to designate *DfGDSL58* as *DfDACX1* (*D. farinosus* DeACetylase on Xylan1) and pursue functional characterization.

Heterologous overexpression of *DfDACX1* in tobacco produced a distinctive phenotype: transgenic plants exhibited significantly increased plant height and basal diameter, accompanied by reduced lignin deposition, decreased xylem width, and lower hemicellulose content (Figure 7). These phenotypic alterations were mirrored at the transcriptional level, with significant downregulation of the xylan synthase gene *NtIRX9* and lignin biosynthesis genes *NtC4H*, *NtCOMT*, and *NtCAD*, while cellulose synthase genes *NtCESA4* and *NtCESA7* remained largely unaffected. This expression pattern is remarkably consistent with the known function of rice *DARX1* as a xylan arabinosyl deacetylase that modulates secondary wall formation [9]. In rice, darx1 mutants exhibited altered xylan acetylation profiles and reduced mechanical strength. Our overexpression results suggest that *DfDACX1* may negatively regulate xylan and lignin biosynthesis pathways, thereby reducing secondary wall thickening and promoting longitudinal growth. This is conceptually analogous to the trade-off between growth and defense/metabolism observed in other plant systems, where modulation of cell wall biosynthesis can reallocate carbon resources toward vegetative growth [13,41,42]. The fact that cellulose content was only marginally affected, while lignin and hemicellulose were significantly reduced, suggests that *DfDACX1* may specifically target the xylan-lignin network in secondary cell walls, rather than globally suppressing all cell wall components.

The functional role of *DfDACX1* in promoting longitudinal growth while suppressing secondary wall thickening has important implications for understanding bamboo’s rapid growth mechanism. Bamboo shoots can elongate at rates exceeding 1 m per day, a process that requires massive cell expansion and cell wall biosynthesis, yet must be coordinated with timely lignification to provide mechanical support [18,19]. Our findings suggest that *DfDACX1* may act as a molecular switch that fine-tunes the balance between cell wall deposition and cell elongation. By modulating xylan deacetylation and downstream lignin biosynthesis, *DfDACX1* could facilitate the rapid expansion of developing internodes while preventing premature or excessive lignification that would constrain growth. This regulatory role is consistent with the emerging paradigm that GDSL lipases function as key modulators of cell wall properties and plant architecture [8]. Notably, the drought-sensitive phenotypes observed in transgenic Arabidopsis overexpressing *DcaGDSL47* from *Dendrobium catenatum* [7] and the drought-tolerant phenotypes conferred by *GhirGDSL26* in cotton [2] highlight the functional diversity of GDSL lipases across species. While those GDSL members are primarily associated with stress responses and cuticle formation, *DfDACX1* appears to have evolved a specialized role in cell wall remodeling during rapid growth, reflecting the unique developmental demands of bamboo. From a biotechnological perspective, our findings may offer a biological strategy complementary to emerging chemical approaches for lignin manipulation. Recent studies have demonstrated the efficient separation of lignin from bamboo biomass using deep eutectic solvents (DESs), which remove adhesive lignin structuration bonds [43,44]. While these chemical methods are effective for post-harvest processing, our work suggests that genetic modulation of lignin deposition in planta—via *DfDACX1*—could reduce the inherent lignin content of bamboo biomass, potentially decreasing the cost and chemical input required for downstream biorefining. This convergence of biological and chemical strategies highlights the potential of combining plant-based genetic improvements with green chemical extraction technologies for sustainable bamboo utilization.

To further contextualize our findings within the bamboo lineage, we surveyed publicly available genomic data from *P. edulis* and *Dendrocalamus latiflorus*, both of which contain GDSL homologs clustering with *DfDACX1* and *OsDARX1*, suggesting evolutionary conservation of this xylan deacetylase subfamily across Bambusoideae. However, functional characterization of these orthologs has not yet been reported in any bamboo species other than *D. farinosus*, underscoring the novelty of our findings and providing a foundation for future comparative studies. Additionally, while our multi-level phenotypic, compositional, and transcriptional evidence strongly supports the role of *DfDACX1* in negatively regulating secondary wall thickening, direct enzymatic activity assays—either in vitro or in planta—would provide more direct biochemical evidence for its xylan deacetylase function. Future studies will focus on recombinant expression and purification of *DfDACX1* protein, establishment of xylan deacetylation activity detection systems, and identification of its specific acetylated substrates. Such investigations will further clarify the substrate specificity and catalytic mechanism of *DfDACX1*, and deepen our understanding of how GDSL lipases regulate cell wall remodeling in bamboo. Future studies incorporating direct mechanical measurements, such as breaking force or flexural bending modulus [45], will also be valuable to definitively ascertain the mechanical properties of *DfDACX1*-overexpressing lines.

## 5. Conclusions

In summary, this study presents the first comprehensive genome-wide characterization of the GDSL gene family in *D. farinosus* and identifies DfDACX1 as a critical regulator of secondary wall synthesis and longitudinal growth. The substantial expansion of the GDSL family in bamboo, particularly within the clade harboring xylan deacetylases, likely reflects adaptive evolution that underpins the remarkable growth rates characteristic of this lineage. Bridging these findings to direct functional validation in bamboo remains a priority for future work. Specifically, future investigations should focus on enzymatic characterization of DfDACX1 to confirm its xylan deacetylase activity and identify its specific substrates, mechanical evaluation to assess the structural integrity of lignin-modified stems, and functional validation through mutant generation in bamboo once stable transformation systems become available [46]. Furthermore, exploring the utility of DfDACX1 for modulating plant architecture or biomass properties in other species may provide valuable strategies for crop improvement and bioenergy applications.

## Data Availability

Data are contained within the article and Supplementary Materials.

## Supplementary Materials

The following supporting information can be downloaded at https://www.mdpi.com/article/10.3390/cells15151427/s1. Supplementary Figure S1: Conserved domain prediction of the DfGDSL protein family. Supplementary Figure S2: Tissue-specific expression analysis of DfGDSL genes (B) and expression level changes in bamboo shoots at different growth stages (A). Supplementary Table S1: NCBI Conserved Domain Database (CDD) Analysis for Protein Sequences. Supplementary Table S2: Amino Acid Sequences for Phylogenetic Analysis. Supplementary Table S3: List of syntenic gene pairs for the GDSL gene family in *D. farinosus*. Supplementary Table S4: Prediction of Subcellular Localization for All *D. farinosus* GDSL Proteins. Supplementary Table S5: Gene structure information of all *D. farinosus* GDSL genes. Supplementary Table S6: List of syntenic gene pairs for the GDSL gene family in *D. farinosus* and rice (*Oryza sativa*). Supplementary Table S7: Detailed data on tissue-specific expression analysis of *DfGDSL genes* and expression level changes in bamboo shoots at different growth stages. Supplementary Table S8: The primers used in this study. Supplementary Table S9: Homologous gene of *DfDACX1* in *P. edulis* and *D. latiflorus*.
