# Supplementary material for: Genome-Wide Identification of the GDSL Gene Family and Functional Validation of DfDACX1 in Secondary Wall Synthesis in Dendrocalamus farinosus
Source: Cells. 2026 Aug 6;15(15):1427. doi: 10.3390/cells15151427 (PMC13465109; doi:10.3390/cells15151427)
Supplement: Supplementary file 1 [file cells-15-01427-s001.zip › Supplementary Figures.pdf]

Genome-Wide Identification of the GDSL Gene Family and Functional Validation of DfDACX1 in Secondary Wall Synthesis in *Dendrocalamus farinosus*

Xin Zhao [1,2,3], Yanwen Zhao [1], Mengqiu Chen [1], Man Tang [1], Zhijian Long [1,2,3], Gang Xu [1,2,3], Ying Cao [1,2,3,*] and Shanglian Hu [1,2,3,*]

[1] College of Life Science and Agri-Forestry, Southwest University of Science and Technology, Mianyang 621000, China
[2] Sichuan Provincial Forestry and Grass Land Key Laboratory for Conservation and Sustainable Utilization of Bamboo Genetic Resources in Southwest of China, Mianyang 621010, China
[3] Key Laboratory of Bamboo Cultivation and Management, National Forestry and Grassland Administration, Mianyang 621000, China
* Correspondence: caoying@swust.edu.cn (Y.C.); hushanglian@swust.edu.cn (S.H.)

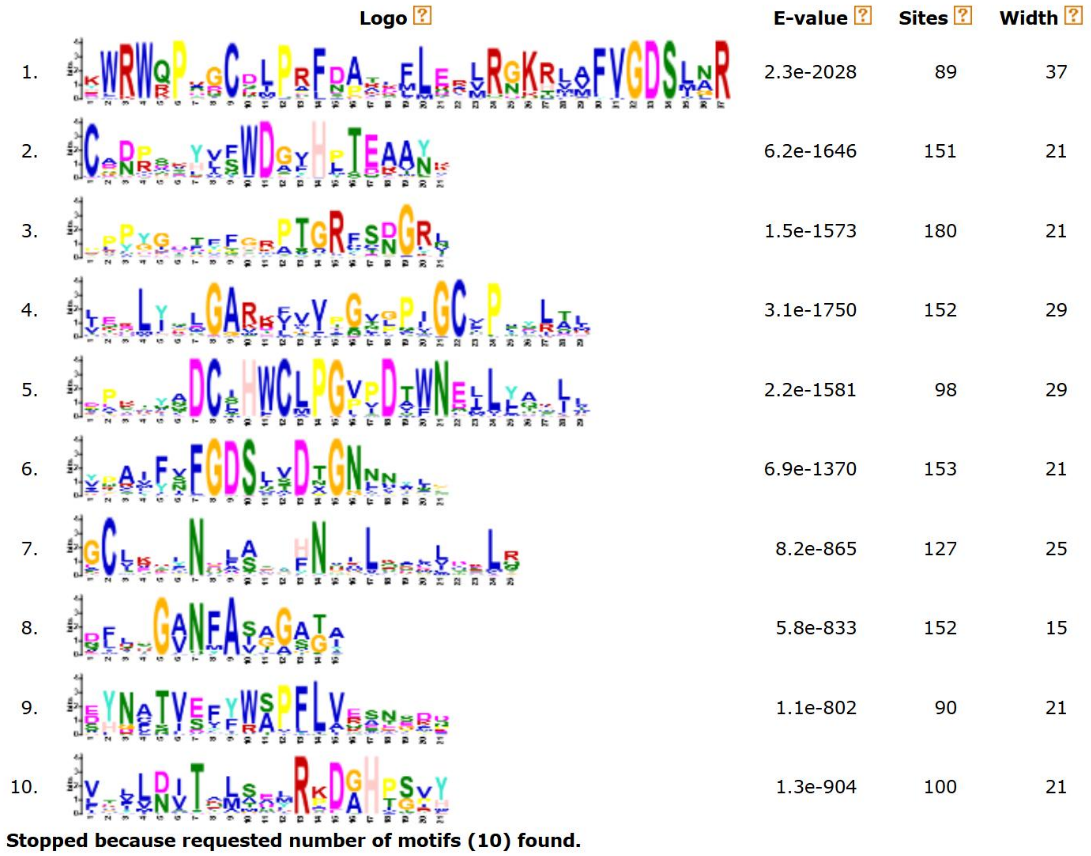

Supplementary Figure S1. Conserved domain prediction of the DfGDSL protein family.

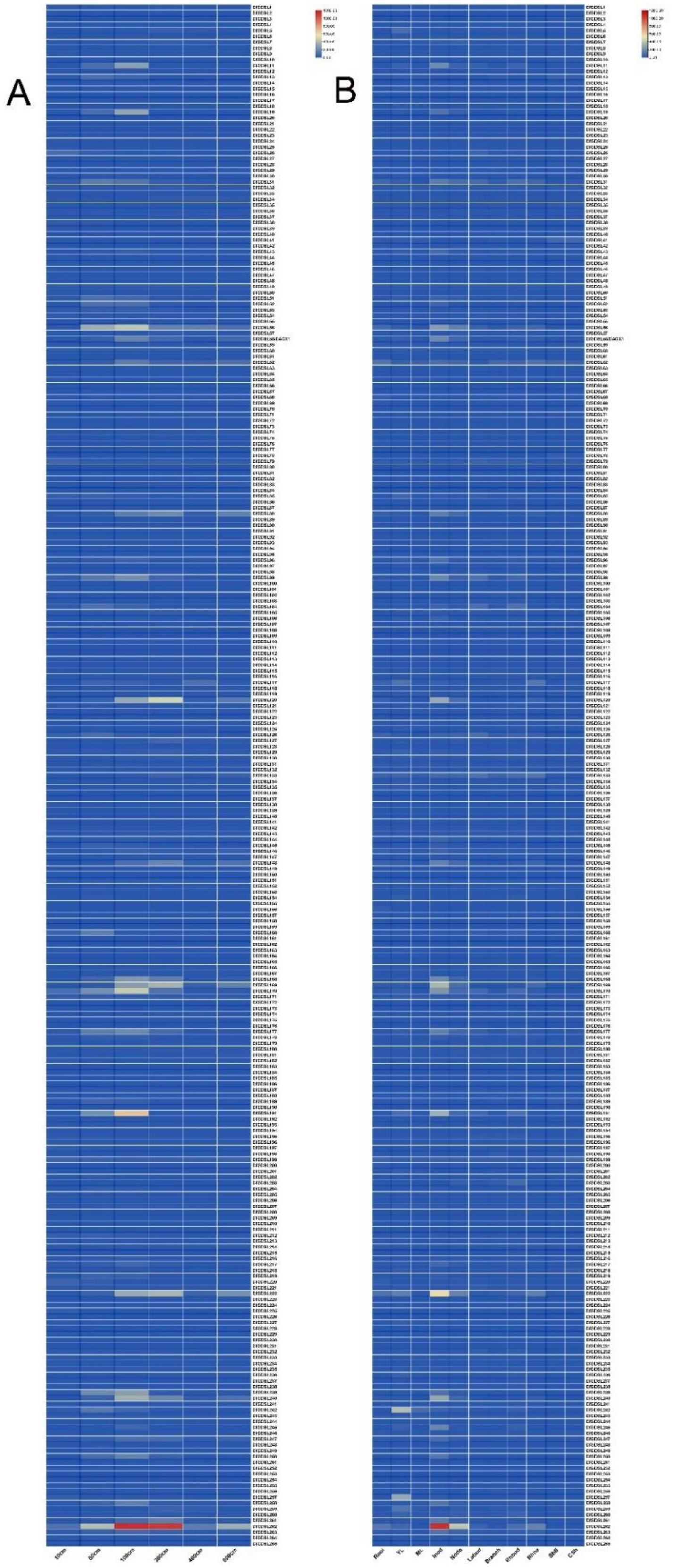

Supplementary Figure S2. Tissue-specific expression analysis of DfGDSL genes (B) and expression level changes in bamboo shoots at different growth stages (A).
